# Antibacterial efficacy of leaf extracts of *Combretum album* Pers. against some pathogenic bacteria

**DOI:** 10.1186/s12906-018-2271-0

**Published:** 2018-07-11

**Authors:** Sunanda Burman, Kuntal Bhattacharya, Devaleena Mukherjee, Goutam Chandra

**Affiliations:** 1 Mosquito, Microbiology and Nanotechnology Research Units, Parasitology Laboratory, Department of Zoology, The University of Burdwan, Golapbag, Burdwan, West Bengal 713104 India; 2 Department of Zoology, Durgapur Government College, JN Avenue, Durgapur, 713214 India

**Keywords:** Antibacterial activity, Pathogenic bacteria, Leaf extracts, *Combretum album*, MIC, Phytochemical screening, FTIR analysis

## Abstract

**Background:**

Plant derived medicines show significant contributions to mankind in treating infections of pathogenic bacteria. Recently plants are used in pharmaceutical industries for novel drug preparations because to ensure efficacy and safety as synthetic antibiotics are threatened for their multidrug resistance. The present study aimed at finding antibacterial potential of aqueous and ethanolic leaf extracts of *Combretum album*.

**Methods:**

Antibacterial activity was evaluated against seven bacterial strains by determining minimum inhibitory concentration (MIC) and zone of inhibition. Diameters of the zone of inhibition were compared with standard antibiotics. Preliminary phytochemical screening was done according to standard protocol. FTIR analysis was performed to identify the general phytochemical groups of compounds in the extract. All experiments were conducted in triplicate and values were expressed as the mean ± standard deviation. One-way analysis of variance (ANOVA) and Tukey tests were performed for statistical justification.

**Results:**

Maximum zones of inhibition were found in case of ethanolic extracts in the following order *Bacillus licheniformis* (MTCC 530) > *Pseudomonas aeruginosa* (MTCC 2453) > *Bacillus subtilis* (MTCC 441) >, *Pseudomonas fluorescens* (MTCC 103) > *Bacillus mycoides* (MTCC 7343) > *Escherichia coli* (MTCC 739) *> Pseudomonas putida* (MTCC 1654) with zone of inhibition of 27.67 ± 0.33 mm diameter in *B. licheniformis* (MTCC 530). Qualitatively, the ethanol extract contains flavonoids, tannins and alkaloids. The results of FTIR analysis confirmed the presence of R-CH2-OH groups, aromatics, C-N stretching amine and NH stretching secondary amine. One way ANOVA and Tukey tests statistically justify the data (*p* ≤ 0.05).

**Conclusions:**

All the tested leaf extracts showed promising antibacterial activity against both gram positive and gram negative bacteria. Phytochemical screening and FTIR analysis revealed the presence of tannins, alkaloids, R-CH2-OH groups, aromatics and flavonoids in ethanolic leaf extract qualitatively and these compounds could be responsible for antibacterial property of leaf extracts of *C. album.*

## Background

Pathogenic bacteria are often showing resistance to antibiotics available in markets for treating bacterial infections due to their indiscriminate uses [1]. In addition their use is limited by a number of factors such as low potency, poor solubility and several other side effects [2]. However, plants, being the inexhaustible source of many drugs, have attracted scientists from ancient times as an alternative source of the commercially available antibiotics. Plants are the producers of various effective secondary metabolites which are the active ingredients of herbal medicines [3, 4]. There are plenty of reports in the literature about the antibacterial activity of plant extracts [5–7]. Plant-derived substances have been used from decades owing to their versatile applications in vector borne diseases, fungal infection, diabetes and many more life threatening ailments [8–12].

According to World Health Organization (WHO) [13] herbal medicines derived from medicinal plants would be the best source for curing diseases and provide primary health care to about three quarters of population of developing countries. Herbal drugs and semi-synthetically produced drugs from botanical sources consisting of nearly 78% of the new drugs approved by the FDA between 1983 and 1994 [14]. Therefore, some herbs, which have been evaluated for antimicrobial activity, may be used to treat a variety of diseases of microbial origin [15, 16]. *Combretum album* Pers. (accepted name of *C. roxburghii* Spreng.) [17] belongs to the ethnobotanically important family Combretaceae. *C. album* is a small sized climber shrub having simple, opposite, oblong to elliptical leaves [18]. *Combretum* has several species and they are known for their several pharmaceutical uses like diarrhoea, inflammation, digestive disorder and some *Combretum* species are also used in diuretic problem [19]. This plant is distributed in India Srilanka, Nepal, Bangladesh, Southern China, Myanmar and along the East Asian countries. Leaves are used in bilious haematuric malarial fever [20] and leaves and bark are known for their antioxidant and cytotoxic activity [21]. Leaves were tested for their antimicrobial properties against some pathogenic bacteria in the present study. Antibacterial effect of *C. album* was aimed to investigate for suggesting a novel source for a probable herbal drug against several bacterial strains.

## Methods

### Plant material

Fresh mature leaves of *Combretum album* Pers. (Family- Combretaceae) were collected from Joypur forest area (23.0540° N, 87.4345° E), Bankura, West Bengal during February to March 2017. The plant was identified by Professor Dr. Ambarish Mukherjee, Department of Botany, The University of Burdwan. The voucher specimen (Voucher number- GCSB05) was submitted to herbarium of Mosquito, Microbiology and Nano-technology Research Units, Parasitology laboratory, Department of Zoology, The University of Burdwan.

### Preparation of crude extract

Fresh leaves were collected and rinsed well in distilled water and excess water was soaked on a paper towel. Clean leaves were grinded by an electrical blender and filtered by Whatman’s no-1 filter paper. The filtrate was considered as the stock crude solution (100% concentration) and stored under refrigeration at 4 °C for further studies.

### Preparation of hot aqueous extract

For preparation of hot aqueous extract 50 g of unspotted leaves were minced and boiled in 500 ml of distilled water for 30 min [22] .The boiled leaves were then kept for 24 h without any disturbance. Resultant extract was filtered and subjected to lyophilisation. Afterwards the powdered extract thus formed was stored at 4 °C for future use.

### Preparation of cold aqueous extract

For preparation of cold aqueous extract 50 g of chopped leaves were soaked into 500 ml of cold water in a stopper bottle in 1:10 ratio [23]. It was left undisturbed for 24 h and filtered through Whatman’s no-1 filter paper. The filtrate thus obtained was lyophilised and then the resultant powder form of extract was kept under refrigeration at 4 °C.

### Preparation of ethanol extract

For preparation of ethanolic extract, fresh and cleaned leaves were shed dried for 14 days. Then 100 g of the dried leaves were finely chopped and extracted in 1000 ml of ethanol through Soxhlet apparatus following 1:10 ratio [23]. The solvent extraction period was set for 8 h per day with total 72 h of extraction period. The extract thus produced was collected from solvent chamber and the excess solvent was evaporated in a rotary evaporator. The residue obtained was stored in a refrigerator for further experiment.

### Microorganisms tested

The tested microorganisms were comprised of three human pathogenic bacterial strains [24] i.e., *Bacillus subtilis* (MTCC 441), *Escherichia coli* (MTCC 739) and *Pseudomonas aeruginosa* (MTCC 2453) and four fish pathogenic bacterial strains [10] namely, *B. licheniformis* (MTCC 530) *B. mycoides* (MTCC 7343), *P. putida* (MTCC 1654), and *P. fluorescens* (MTCC 103) of clinical importance. The strains were obtained from Mosquito, Microbiology and Nanotechnology Research Units, Parasitology Laboratory, The University of Burdwan. All the bacterial strains were cultured in nutrient broth (Hi-Media, M002) at 37 °C and were maintained on nutrient agar slants at 4 °C.

### Determination of antibacterial assay

#### Inoculum standardization

All bacterial strains were inoculated in Müeller-Hinton broth (pH 7.4.) for about 16 h. The concentration of the suspensions was adjusted to 0.5 (optical density) with the help of spectrophotometer.

#### Assay of antibacterial activity using agar well diffusion method

Antimicrobial activity of the crude and solvent extracts was determined by the Agar well diffusion method [23]. The 20 ml of sterilized Nutrient Agar was poured into sterile petri plates. After solidification, 100 μl of standardized inoculate of each isolate was inoculated on Nutrient agar plates by using sterilized spreaders. The wells were punched over the agar plates using sterile gel puncher of 6 mm diameter. 100 μl of each extract was poured in to separate wells. Extracts were dissolved in 1% (*v*/v) dimethylsulphoxide (DMSO) which was taken as negative control for solvent extract. Four different concentrations of aqueous and ethanolic extracts (50, 100, 200 and 400 μg/ml) were tested. Plates were incubated at 37 °C for 24 h. Triplets of the experiment were maintained for each bacterial strain to ensure reliability. After incubation the diameter of circular inhibitory zones formed around each well were measured in mm and recorded.

#### Antibiotic susceptibility test

Antibiogram was done by disc diffusion method using standard antimicrobial sensitivity testing antibiotics obtained from Hi-Media Laboratories Limited, Bombay were use. Agar plates were prepared and the test organisms were swabbed over the surface of the solidified agar plates using sterile swab spreader. Then standard antibiotic discs were impregnated on the inoculated agar plates and incubated for 24 h at 37 °C. This was followed by measurement of zone of inhibition formed by the test bacteria around the standard antibiotic discs [4].

#### Minimum inhibitory concentration (MIC)

Standard methods of antimicrobial susceptibility test approved by the National Committee for Clinical Laboratory Science (NCCLS) [25] for testing conventional drugs cannot be exactly applicable to plant extracts. Hence, modifications have been made for evaluating antibacterial activity of active principles of plant origin. Minimum inhibitory concentration (MIC) was determined for each extract showing antimicrobial activity against the test isolates using broth dilution method with slight modifications [25, 26]. Each culture was diluted in Müeller-Hinton broth. The concentration of test cultures was adjusted to turbidity of 0.5 McFarland standards. Equal volume (0.5 ml) of each extract (by serial dilutions from the stock solution of cold aqueous, hot aqueous and ethanol extract) and nutrient broth were mixed in the test tubes. Final concentrations of the three extracts in each of the test tube were in the range of 40, 35, 30, 25, 20, 10, 5, 2.5, 1.25 and 0.625 μg/ml. Specifically 0.1 ml of standardized inoculum (5 × 10^5^ CFU/ml) was added to each tube. All the tubes were incubated for 24 h at 37 °C. After incubation growth were examined and data were recorded. The MIC values were taken as the lowest concentration of the extracts showing no growth in the tube. Experiment was repeated thrice for authentication of the data.

#### Phytochemical screening

Phytochemical analysis was done using the protocols by Harbone and Sofowora [27, 28] for testing the presence of tannins, saponins, steroids, terpenoids, glycosides, alkaloids, anthroquinones and flavonoids in ethanolic extract of *C. album* leaves.

#### Fourier transform infrared spectrophotometer (FTIR) analysis

Fourier Transform Infrared Spectrophotometer (FTIR) is the most reliable tool for identifying the types of chemical bonds (functional groups) in the plant extracts. Dried powder of ethanol extract of *C. album* leaves was used for FTIR analysis. For preparing translucent sample discs 10 mg of the dried extract powder was encapsulated in 100 mg of KBr pellet by using hydrolic press. KBr pellet was used as control. The pellets were loaded in FTIR spectrometer (Jasco, FT/IR- 4700), with a scan range from 400 to 4500 cm-^1^ in order to determine the functional groups in the ethanolic extract.

#### Statistical analysis

The data were analysed by using MS Excel 2007 and presented as mean ± SD of three replicates. One-way analysis of variance (ANOVA) and Tukey tests were performed by using ‘Stat plus 2009 professional’ trial version software to determine significant group differences and means were considered as statistically significant if *p* < 0.05.

## Results

**Fig. 1:**
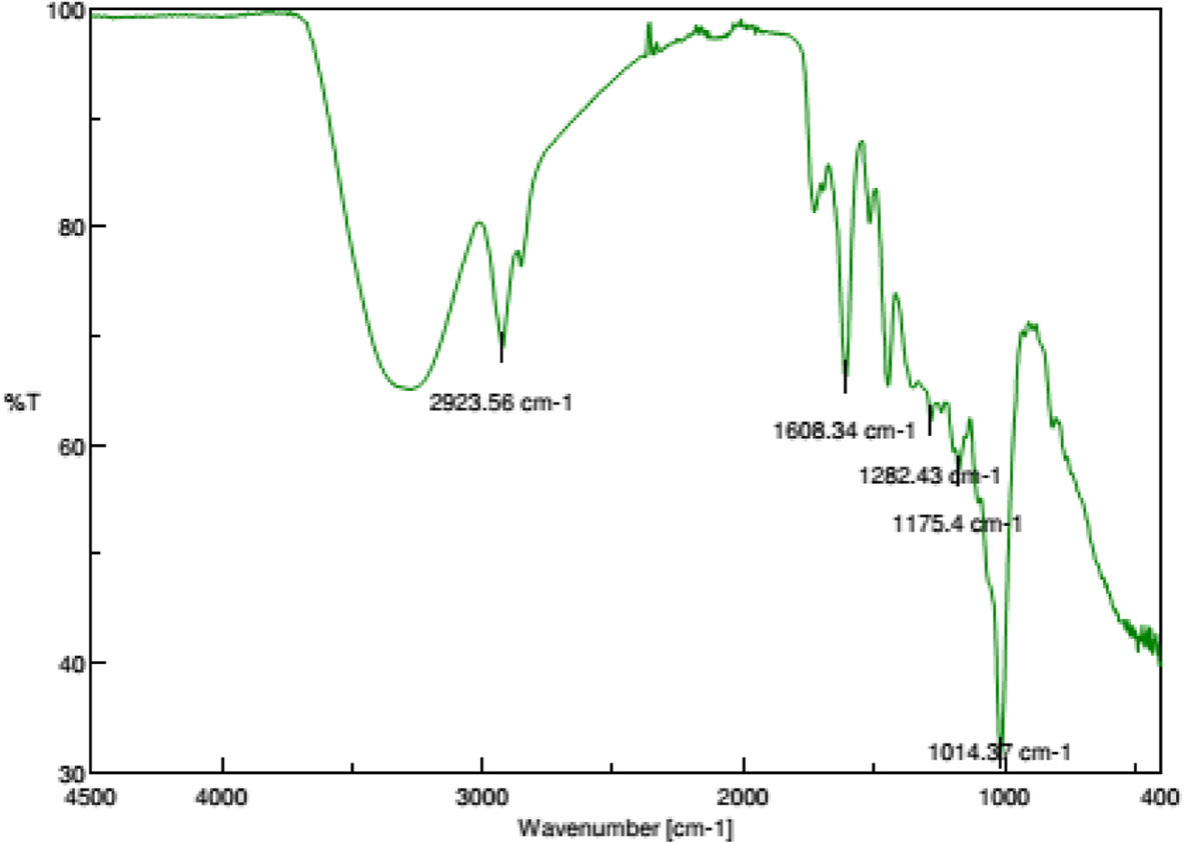
Fourier transform infrared spectroscopy (FTIR) analyses ethanolic leaf extract of *Combretum album*

Leaf extracts of *C. album* showed significant antibacterial activity against the seven tested pathogenic bacteria. In this experiment crude, hot aqueous, cold aqueous and ethanolic extract showed inhibitory property towards all bacterial isolates with *B. licheniformis* (MTCC 530) being most susceptible to the ethanolic extract (400 μg/ml) with inhibition zone of 27.67 ± 0.33 mm followed by *P. aeruginosa* (MTCC 2453)*, B. subtilis* (MTCC 441)*, P. fluorescens* (MTCC 103)*, B. mycoides* (MTCC 7343)*, E. coli* (MTCC 739)*,* and *P. putida* (MTCC 1654) with inhibition zones of 27.33 ± 0.33 mm, 27.00 ± 0.00 mm, 26.67 ± 0.33 mm, 24.67 ± 0.33 mm, 23.67 ± 0.33 mm and 21.33 ± 0.33 mm of diameter respectively. Whereas negative controls i.e., distilled water and DMSO did not show any inhibitory effect to the growth of all tested bacteria (Table 1). Results of antibiogram in this investigation depicted that all the bacterial isolates were resistant to standard antibiotics like Ampicillin (10 μg/disc) and Penicillin G (10 μg/disc) and also Amoxicillin (30 μg/disc) had very little effect against them*.* Susceptibility of all the gram negative bacteria to Gentamycin (30 μg/disc), and both gram negative and gram positive bacteria to Tetracycline (10 μg/disc) and Erythromycin (5 μg/disc) was higher among the respective tested antibiotics and the bacterial strains showed differential susceptibility to different antibiotics (Table 1). *E. coli* (MTCC 739) was most susceptible to Gentamycin (30 μg/disc) while growth of *B. subtilis* (MTCC 441) was inhibited with maximum inhibition zone by Erythromycin (5 μg/disc) and Tetracycline (10 μg/disc) (Table 1). It was also observed from the result that antibacterial activity of the extracts increased with the increase in the concentration used. MIC was tested with serial dilution of the leaf extracts and the results indicated a very low concentrations were effective (Table 2) against the tested bacterial strains. Analysis of variance table (One way ANOVA) and Tukey test for differences between means demonstrated statistical significance of the data (*p* ≤ 0.05). Phytochemical analysis revealed the presence of secondary metabolites like flavonoids, tannins and alkaloids (Table 3) in the ethanolic extract of *C. album.* FTIR analysis (Fig. 1) of the ethanolic leaf extract revealed major bands at 2923.56 cm^− 1^, 1608.34 cm^− 1^, 1282.43 cm^− 1^, 1175.4 cm^− 1^ and 1014.37 cm^− 1^. The bands at 3400–3200 cm^− 1^ implied to the OH stretching hydrogen bonded broad peak and C-O stretch at bands at 1075–1000 cm^− 1^ confirming the presence of R-CH2-OH groups (Alcohols). The bands at 1040–995 cm^− 1^ were due to CH bending in plane H bend 730–680 cm^− 1^ out of plane ring bending associated with the presence of aromatics in the extract. C-N stretching amine and NH stretching secondary amine were indicated by bands at 1146–1132 cm^− 1^ and 3500–3300 cm^− 1^. Bands at 1700–1680 cm^− 1^, 1320–1211 cm^− 1^ and 960–875 cm^− 1^ unveiled the presence of C=O stretching, C-O stretching and O-H deformation with broad out-of-plane OH…O deformation corresponding to Ph-COOH (carboxylic acids) respectively.

**Table 1:** Antibacterial Bioassay of different extracts of leaves of *Combretum album* and susceptibility test of some standard antibiotics

Bacterial strains	Diameter of inhibition zone(mm)															Antibiotics ^a^					
	Crude extract	Cold aqueous extract (μg/ml)				Hot aqueous extract (μg/ml)				Ethanol extract (μg/ml)				Distilled water	DMSO	Ampicillin	Gentamycin	Penicillin G	Erythromycin	Tetracycline	Amoxicillin
	0.1%	50	100	200	400	50	100	200	400	50	100	200	400	100 μl/well	100 μl/well	(10 μgdisc)	(30 μg/disc)	(10 μg/disc)	(5 μg/dics)	(10 μg/disc)	(30 μg/disc)
Human pathogens	100 μl/well																				
*B. subtilis* (MTCC 441)	20.33 ± 0.33	21.33 ± 0.33	23.00 ± 0.58	24.33 ± 0.33	26.00 ± 0.58	18.00 ± 0.58	18.67 ± 0.33	19.67 ± 0.33	22.67 ± 0.33	23.67 ± 0.33	24.33 ± 0.33	25.67 ± 0.33	27.00 ± 0.00	0.00 ± 0.00	0.00 ± 0.00	0.00 ± 0.00	NA	0.00 ± 0.00	29.67 ± 0.33	29.00 ± 0.00	15.33 ± 0.33
*E. coli* (MTCC 739)	21.00 ± 0.00	16.33 ± 0.33	17.00 ± 0.00	18.33 ± 0.33	21.67 ± 0.88	16.67 ± 0.33	17.67 ± 0.33	18.33 ± 0.33	22.33 ± 0.67	17.33 ± 0.67	20.00 ± 0.58	21.67 ± 0.33	23.67 ± 0.33	0.00 ± 0.00	0.00 ± 0.00	NA	23.67 ± 0.88	NA	23.00 ± 0.58	24.00 ± 0.00	NA
*P. aeruginosa* (MTCC 2453)	22.00 ± 0.58	16.67 ± 0.33	17.67 ± 0.67	19.33 ± 0.33	21.00 ± 0.58	16.33 ± 0.33	17.33 ± 0.67	18.67 ± 0.33	20.33 ± 0.33	20.00 ± 0.58	22.33 ± 0.33	24.33 ± 0.33	27.33 ± 0.33	0.00 ± 0.00	0.00 ± 0.00	NA	27.33 ± 0.67	NA	23.33 ± 0.67	14.33 ± 0.67	NA
Fish pathogens																					
*B. licheniformis* (MTCC 530)	22.33 ± 0.33	17.67 ± 0.33	19.00 ± 0.00	19.67 ± 0.33	21.00 ± 0.00	18.67 ± 0.33	19.00 ± 0.00	20.33 ± 0.33	21.00 ± 0.58	22.00 ± 0.58	22.33 ± 0.33	25.33 ± 0.33	27.67 ± 0.33	0.00 ± 0.00	0.00 ± 0.00	0.00 ± 0.00	NA	0.00 ± 0.00	18.66 ± 0.33	22.00 ± 0.58	17.33 ± 0.33
*B. mycoides* (MTCC 7343)	21.33 ± 0.33	17.67 ± 0.33	20.33 ± 0.33	21.00 ± 0.58	22.33 ± 0.33	16.67 ± 0.33	18.33 ± 0.33	19.67 ± 0.33	22.00 ± 0.58	19.33 ± 0.67	20.67 ± 0.33	22.00 ± 0.58	24.67 ± 0.33	0.00 ± 0.00	0.00 ± 0.00	0.00 ± 0.00	NA	0.00 ± 0.00	26.00 ± 0.58	15.00 ± 0.00	12.00 ± 0.58
*P. putida* (MTCC 1654)	16.67 ± 0.33	16.00 ± 0.58	17.00 ± 0.58	18.00 ± 0.58	20.33 ± 0.33	16.67 ± 0.33	17.67 ± 0.33	19.00 ± 0.58	20.00 ± 0.58	16.33 ± 0.33	18.00 ± 0.00	19.67 ± 0.33	21.33 ± 0.33	0.00 ± 0.00	0.00 ± 0.00	NA	26.67 ± 0.33	NA	25.33 ± 0.33	14.33 ± 0.33	NA
*P. fluoroscens* (MTCC 103)	22.00 ± 0.00	17.33 ± 0.67	19.67 ± 0.33	21.67 ± 0.67	23.33 ± 0.33	21.00 ± 0.58	22.33 ± 0.67	23.00 ± 0.58	24.00 ± 0.58	21.33 ± 0.33	23.33 ± 0.33	25.00 ± 0.00	26.67 ± 0.33	0.00 ± 0.00	0.00 ± 0.00	NA	23.67 ± 0.33	NA	29.67 ± 0.33	14.00 ± 0.88	NA

^a^ Antibiotics were used based on their spectrum of activity i.e., their efficacy against gram positive, gram negative or both

**Table 2:** Minimum Inhibitory Concentration of different leaf extracts of *C. album*

Bacterial strains	Minimum inhibitory concentration (μg/ml)		
	Cold water extract	Hot water extract	Ethanol extract
*B. subtilis* (MTCC 441)	25.00 ± 0.00	25.00 ± 0.00	15.00 ± 0.00
*P. aeruginosa* (MTCC 2453)	20.00 ± 0.00	15.00 ± 0.00	5.00 ± 0.00
*E coli* (MTCC 739)	30.00 ± 0.00	20.00 ± 0.00	15.00 ± 0.00
*B. licheniformis* (MTCC 530)	20.00 ± 0.00	15.00 ± 0.00	5.00 ± 0.00
*B. mycoides* (MTCC 7343)	35.00 ± 0.00	25.00 ± 0.00	20.00 ± 0.00
*P. putida* (MTCC 1654)	30.00 ± 0.00	20.00 ± 0.00	10.00 ± 0.00
*P. fluoroscens* (MTCC 103)	25.00 ± 0.00	25.00 ± 0.00	15.00 ± 0.00

**Table 3:** Secondary metabolites obtained in *Combretum album* leaves

Phytochemicals	Ethanol extract
Tannins	+Ve
Saponins	-Ve
Steroids	-Ve
Terpenoids	-Ve
Glycosides	-Ve
Alkaloids	+ Ve
anthraquinones	-Ve
Flavonoids	+Ve

## Discussion

Presence of effective phytochemicals in higher plants has been known for their antibacterial activity. Effectiveness of the phytoconstituents may be related to the solvents used for their extraction. In Ayurveda extraction with water and ethanol is well known because of their nontoxic property and edibility. Mukhtar et al. [29] reported the use of aqueous and ethanolic medicinal plant extract for their antimicrobial activity. Similar effects of ethanolic extract observed in the works of many researchers [30–32].

Modes of action of plant extracts that are distinct from those of the antibiotics in current use, suggesting that cross-resistance with agents already in use is negligible [33]. All of the extracts showed a linear antibacterial efficacy to all tested bacteria and therefore supported the statement that there is no correlation between the susceptibility shown by *C. album* extracts and the resistance to any other standard antibiotics tested. MIC (Table 2) is very low in comparison to results of many medicinal plants observed in several other research works [10, 34]. From the results of MIC and zone of inhibition values and their competition to that of the standard antibiotics it can be concluded that the leaf extracts are considered to have a broad spectrum antibacterial activity against both gram positive and gram negative bacteria. It was also evident from the result that antibacterial activity increased with the increase in concentration of all extracts in all tested bacteria (Table 1).

Kumar has reported antibacterial activity of ethanol extract of flower of *Combretum indicum*, which showed highest inhibition zone (18 mm) against *Staphylococcus aureus* whereas *P. aeruginosa* showed intermediate sensitivity (16 mm) at 100 mg/ml concentration [35]. Oghenejobo et al. [36], while tested ethanolic extract of leaves of *Combretum racemosum* against *S. aureus*, *P. aeruginosa*, *Proteus vulgaris*, *E. coli* and *Salmonella typhi*, highest inhibition zone (25 mm) was observed against *S. aureus* at 31.25 mg/ml concentration. Present study indicates that *C. album* exhibited much higher antibacterial activity in lesser concentration comparative to *C. indicum* and *C. racemosum*. Panda et al. [37] reported positive antibacterial effectiveness of aqueous and methanolic bark and leaf extract of *C. album* against some bacteria such as *Staphylococcus aureus, Shigella dysentriae, S. flexneri* and *Vibrio cholera.* Moreover our work was conducted against different bacteria and more significant results were found using aqueous and ethanolic extracts. The compounds responsible for this antibacterial activity though not investigated, preliminary phytochemical analysis of the ethanolic extract exposed the presence of tannin, alkaloid and flavonoid compounds and the presence of alcoholic, amine, carboxylic acids and aromatic groups was revealed by FTIR analysis which supports these findings. According to Hideyuki et al. [38] and Meng et al. [39] flavonoids and tannins may contribute to antibacterial properties. Thus it can be inferred that antibacterial properties of *C. album* leaf may be attributed to the individual or combined effect of the above mentioned chemical groups [40, 41]. The antibacterial potency of the plants is thought to be due to functional groups like alcoholic, aromatic, amine and carbo-acids present in tannins, alkaloids and flavonoids detected by FTIR analysis which can be extracted from this plant and used in herbal drug preparations valuable for the treatment of many bacteria borne diseases and this should be fully explored in proper approach. This finding could also emphatically contribute to increase the therapeutic value of the particular chemicals in the plant which can be acted as antibacterial agents. The findings of the present study offer the ethnomedical use of this plant by the pharmaceutical industries. Further studies are needed to pin point the active ingredient responsible for antibacterial activity.

## Conclusions

Increase in resistance to commercially available antibiotics projects major dilemma in the treatment of bacterial infections throughout the world. Based on the above investigation it can be concluded that leaves of *C. album* can be a potential source for herbal drug preparations against pathogenic bacteria. In Future phytochemical group wise screening and isolation of bio-active compounds will be further investigated to develop as a new therapeutic agent to fight infectious diseases.

## Abbreviations

DMSO: Dimethyl Sulfoxide
FTIR: Fourier Transform Infrared Spectrophotometer
MIC: Minimum inhibitory concentration
SD: Standard Deviation
